# Murine *Borrelia *arthritis is highly dependent on ASC and caspase-1, but independent of NLRP3

**DOI:** 10.1186/ar4090

**Published:** 2012-11-13

**Authors:** Marije Oosting, Kathrin Buffen, Subbarao RK Malireddi, Patrick Sturm, Ineke Verschueren, Marije I Koenders, Frank L van de Veerdonk, Jos WM van der Meer, Mihai G Netea, Thirumala-Devi Kanneganti, Leo AB Joosten

**Affiliations:** 1 Department of Medicine, Radboud University Nijmegen Medical Centre, Geert Grooteplein zuid 8, Nijmegen, 6525GA,The Netherlands; 2 Nijmegen Institute of Infection, Inflammation and Immunity (N4i), Radboud University Nijmegen Medical Centre, Geert Grooteplein zuid 8, Nijmegen, 6525GA,The Netherlands; 3 Department of Immunology, St. Jude Children's Research Hospital, 262 Danny Thomas Place, Memphis, TN 38105, USA; 4 Department of Microbiology, Radboud University Nijmegen Medical Centre, Geert Grooteplein zuid 10, Nijmegen, 6525GA, The Netherlands; 5 Department of Rheumatology, Radboud University Nijmegen Medical Centre, Nijmegen, Geert Grooteplein zuid 10, 6525GA, The Netherlands

## Abstract

**Introduction:**

The protein platform called the NOD-like-receptor -family member (NLRP)-3 inflammasome needs to be activated to process intracellular caspase-1. Active caspase-1 is able to cleave pro-Interleukin (IL)-1β, resulting in bioactive IL-1β. IL-1β is a potent proinflammatory cytokine, and thought to play a key role in the pathogenesis of Lyme arthritis, a common manifestation of *Borrelia burgdorferi *infection. The precise pathways through which *B. burgdorferi *recognition leads to inflammasome activation and processing of IL-1β in Lyme arthritis has not been elucidated. In the present study, we investigated the contribution of several pattern recognition receptors and inflammasome components in a novel murine model of Lyme arthritis.

**Methods:**

Lyme arthritis was elicited by live *B. burgdorferi*, injected intra-articularly in knee joints of mice. To identify the relevant pathway components, the model was applied to wild-type, NLRP3-/-, ASC-/-, caspase-1-/-, NOD1-/-, NOD2-/-, and RICK-/- mice. As a control, TLR2-/-, Myd88-/- and IL-1R-/- mice were used. Peritoneal macrophages and bone marrow-derived macrophages were used for *in vitro *cytokine production and inflammasome activation studies. Joint inflammation was analyzed in synovial specimens and whole knee joints. Mann-Whitney *U *tests were used to detect statistical differences.

**Results:**

We demonstrate that ASC/caspase-1-driven IL-1β is crucial for induction of *B. burgdorferi-*induced murine Lyme arthritis. In addition, we show that *B. burgdorferi-*induced murine Lyme arthritis is less dependent on NOD1/NOD2/RICK pathways while the TLR2-MyD88 pathway is crucial.

**Conclusions:**

Murine Lyme arthritis is strongly dependent on IL-1 production, and *B. burgdorferi *induces inflammasome-mediated caspase-1 activation. Next to that, murine Lyme arthritis is ASC- and caspase-1-dependent, but NLRP3, NOD1, NOD2, and RICK independent. Also, caspase-1 activation by *B. burgdorferi *is dependent on TLR2 and MyD88. Based on present results indicating that IL-1 is one of the major mediators in Lyme arthritis, there is a rationale to propose that neutralizing IL-1 activity may also have beneficial effects in chronic Lyme arthritis.

## Introduction

Lyme disease is a complex infectious disease, caused by spirochetes of the *Borrelia burgdorferi *sensu lato family. The initial host response toward *Borrelia *is mediated by the innate immune system through recognition by pattern recognition receptors (PRRs) [1-3].

Toll-like receptor 2 (TLR2) recognizes *Borrelia *species. Cells from TLR2-deficient mice show decreased cytokine production after exposure to *Borrelia *species [4], and infection with live *B. burgdorferi *in these mice results in up to 100-fold more spirochetes in their joints [5]. Cells of humans bearing a single nucleotide polymorphism (SNP) in their TLR2 gene show reduced cytokine production when exposed to *Borrelia-*derived antigens [6]. Furthermore, we and others have found that TLR1/2, but not TLR2/6, heterodimers are essential for *B. burgdorferi-*dependent cytokine production [7,8]. The crucial role for a TLR-mediated pathway was further underlined by studies using myeloid differentiation factor 88 (MyD88) gene-deficient mice [9,10]. When Myd88-deficient mice were injected with live *B. burgdorferi*, highly elevated spirochetal burden was found in several organs of the mice, indicating the pivotal role of Myd88 in innate host defense against *Borrelia *[11].

*B. burgdorferi *is recognized by the intracellular receptor nucleotide-binding oligomerization domain-containing protein 2 (NOD2), a member of the NOD-like receptor (NLR) family. Recently, it was demonstrated that NOD2 is needed for optimal cytokine production after *B. burgdorferi *stimulation in mice, but does not affect the spirochetal burden [12]. Cells from humans bearing the NOD2 frameshift mutation produced less IL-1β when exposed to *B. burgdorferi*, indicating that this PRR is also important in Lyme disease [3].

The proinflammatory cytokine interleukin (IL)-1β is known to play a major role in the pathogenesis of Lyme arthritis [13-15]. Synthesis of its inactive precursor pro-IL-1β is initiated by signals induced through PRRs [16], processing of pro-IL-1β to yield the active cytokine requires cleavage by caspase-1 [17]. In turn, caspase-1 activation needs assembly of a protein platform known as the inflammasome, of which the NLR-family member NLRP3 is the most studied [18].

In the present study, we explored the signaling pathways involved in recognition of *B. burgdorferi *by immune cells and their effect on the induction of cytokines. We investigated the two major recognition pathways for *B. burgdorferi*, TLR2-MyD88 and NOD2-serine-threonine protein kinase with a caspase activation and recruitment domain (RICK) in the induction of Lyme arthritis. In addition, the role of components of the NLRP3 inflammasome for *Borrelia-*induced Lyme arthritis was explored.

## Materials and methods

### *Borrelia burgdorferi *cultures

*B. burgdorferi *ATCC strain 35210, was cultured at 33°C in Barbour-Stoenner-Kelley (BSK)-H medium (Sigma-Aldrich, St Louis, MI, USA) supplemented with 6% rabbit serum. Spirochetes were grown as described by Oosting *et al *[19].

### Animals

IL-1R knockout (KO) mice were from Jackson Laboratories (Bar Harbor, MA, USA) (B6.129S7-*Il1r1^tm1Imx^*/J). Female wild-type (WT) (C57Bl/6J) and KO mice between eight and ten weeks of age were used. The mice were fed sterilized laboratory chow (Hope Farms, Woerden, The Netherlands) and water *ad libitum*. The protocol was approved by the Ethics Committee on Animal Experiments of the Radboud University Nijmegen Medical Centre (RU-DEC-2011-013). MyD88-/-, TLR2-/-, NOD1-/-, NOD2-/-, RICK-/-, ASC-/-, NLRP3-/-, and caspase-1-/- mice were bred and maintained in the St. Jude Children's Research Hospital, Memphis, TN, USA, as previously described [4,20-25]. Animal studies were conducted under protocols approved by St. Jude Children's Research Hospital Committee on Use and Care of Animals.

### *In vitro *cytokine production

Bone marrow was isolated according to Oosting *et al *[19]. For stimulation of bone marrow-derived macrophages (BMDMs) and cytokine measurements, see Additional file 1.

### Western blot

Western blot analysis was performed according to the procedure described by Oosting *et al. *[19].

### Induction of *Borrelia*-induced joint inflammation and histology

Joint inflammation was induced by intra-articular (i.a.) injection of 1 × 10^7 ^live *B. burgdorferi *in 10 μL of PBS into the right knee joint of naïve or KO mice. Four hours after i.a. injection, mice were sacrificed and synovial specimens were isolated. After 24 h, knee joints were removed for histology. Before removal of the joints, macroscopic score of the thickness of the joints (without skin) was performed ranging from no swelling (score is 0) or very severely swollen joints (score is referred to as 3). Whole knee joints were removed and fixed in 4% formaldehyde for seven days before decalcification in 5% formic acid and processing for paraffin embedding. Histology of 7 μM knee sections was performed as described before [19].

### RNA isolation and real-time quantitative PCR

RNA from mouse cells was isolated using TRIzol reagent (Invitrogen, Carlsbad, CA, USA) following the manufacturer's instructions. Isolated RNA was reversed transcribed into complementary DNA using iScript cDNA synthesis kit (Bio-Rad Laboratories, Veenendaal, The Netherlands). See Additional file 1 for quantitative real-time PCR.

### Isolation of patella biopsies and patella washout assays

After resection of the patella with surrounding tissue, biopsies for mRNA expression assays were isolated using 3 mm disposable biopsy punches (Miltex, Integra, Germany) and immediately after isolation frozen in liquid nitrogen. Samples were stored at -80°C until RNA was extracted according to the method described above. Before RNA isolation, biopsies were lysed using the MagNALyser (Roche Applied Science, Penzberg, Germany). Patella washout assays were performed as described before [19].

### Statistical analysis

The data are expressed as mean ± SEM. Differences between experimental groups were tested using the two-sided Mann-Whitney *U *test performed on GraphPad Prism version 4.0 software (GraphPad, San Diego, CA, USA). *P *values of ≤0.05 were considered significant.

## Results

### Induction of murine Lyme arthritis by intra-articular injection of live *B. burgdorferi*

Infection of C3H/HeN mice is a standard model for Lyme arthritis. C3H/HeN mice are highly susceptible to develop severe Lyme arthritis upon intradermal injection with *Borrelia*. C57Bl/6 mice are known to develop only mild symptoms caused by *Borrelia *species [26]. To induce murine Lyme arthritis in C57Bl/6 mice, we used several application routes, ranging from intraperitoneal, intravenous, and intradermal in the lower back. However, none of these injection routes resulted in the development of detectable arthritis in WT C57/Bl6 mice (data not shown). In addition, we performed studies with dose ranges up to 1 × 10^7 ^spirochetes per injection. No signs of *Borrelia-*induced joint inflammation were seen in the C57/Bl6 mice (data not shown).

**Figure 1:**
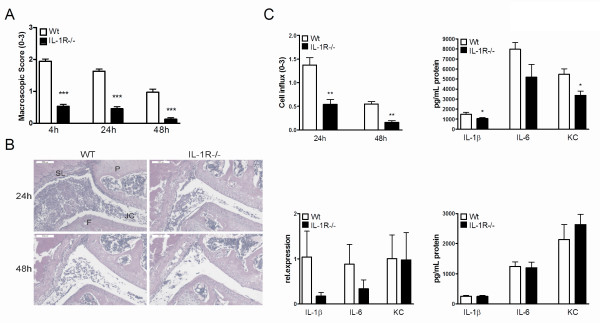
**Murine Lyme arthritis is IL-1-dependent**. **(A) **Macroscopic score of the knees in either wild-type (WT) (white bars), or IL-1 Receptor-/- mice (black bars). After 4, 24, and 48 hours of intra-articular (i.a.) injection of 1 × 10^7 live *B. burgdorferi*, at least 10 knees per group. Data are mean ± SEM from eight animals in each group; ***P <0.0001; Mann-Whitney *U *test, two-tailed. **(B) **Murine Lyme arthritis in WT, or IL-1R-/- mice. Histology (H&E staining) 24, and 48 hours after i.a. injection of *B. burgdorferi *in knee joints. 200× magnification; P, patella; F, femur; JC, joint cavity; SL, synovial lining. Scale bar represents 100 μM. **(C) **Upper left: Scored cell influx after 24 and 48 hours of i.a. injection with *B. burgdorferi*. Upper right and lower left: 4 hours after i.a. injection of 1 × 10^7 live *B. burgdorferi *in 10 μL of PBS, patellae were cultured for 1 h and IL-1β, IL-6 and keratinocyte-derived chemokine (KC) protein levels and mRNA expression levels were measured using Luminex and qPCR, respectively. Lower right: After 24 hours of infection, 1 × 10^5 peritoneal macrophages were stimulated for 24 hours with live *B. burgdorferi*. White bars represent cytokine induction by WT mice, black bars are IL-1R gene-deficient mice, at least five animals/group. *P <0.05, **P <0.01; Mann-Whitney *U *test, two-sided.

It has been demonstrated in patients with Lyme arthritis that *Borrelia *spirochetes were detected in synovial fluid or tissue using either PCR or culture [27,28]. Therefore, we injected live *Borrelia *directly into knee joints of C57Bl/6 mice to mimic the clinical practice of patients with active Lyme arthritis. We were able to induce joint inflammation resulting in joint swelling and cell influx into the joint cavity up to day 7 after i.a. injection of spirochetes (Figure 1A/B). Using this novel model of Lyme arthritis we could address the goal of the current study, to investigate the upstream mediators of *Borrelia burgdorferi-*induced activation of the inflammasome and the contribution of individual components of the inflammasome in Lyme arthritis.

### Murine Lyme arthritis is strongly dependent on IL-1 production

IL-1 was shown already to play an important role in the pathogenesis of Lyme disease, but the role in the development of murine Lyme arthritis has never been described so far [15]. To corroborate the role of IL-1 in the induction of our novel murine model of Lyme arthritis, IL-1R-deficient mice were injected intra-articularly with live *B. burgdorferi*. Compared to WT mice, IL-1R-/- mice exhibited significantly reduced joint swelling at early (4 h) and late (48 h) time points (Figure 1A). This was reflected by histology: IL-1R gene-deficient mice displayed a considerable reduction in the numbers of inflammatory cells in the joint cavity when compared to WT mice (Figure 1B). At 24 h and 48 h after induction of Lyme arthritis, the cell influx was decreased in IL-1R-/- mice (Figure 1C, upper left). In these mice, significantly reduced protein concentrations of IL-1β and the keratinocyte-derived chemokine (KC) were found in patella washouts (Figure 1C, upper right). Synovial tissue explants of IL-1R-/- mice showed less IL-1β, and IL-6 mRNA expression, 4 h after induction of Lyme arthritis (Figure 1C, lower left). KC mRNA expression was found to be similar between WT and IL-1R-/- mice (Figure 1C, lower left). No differences between WT and IL-1R KO mice were observed in IL-1β, IL-6, and KC production of peritoneal macrophages stimulated with *B. burgdorferi *(Figure 1C, lower right).

### *Borrelia*-induced IL-1β production is NOD1/2 and RICK independent

**Figure 2:**
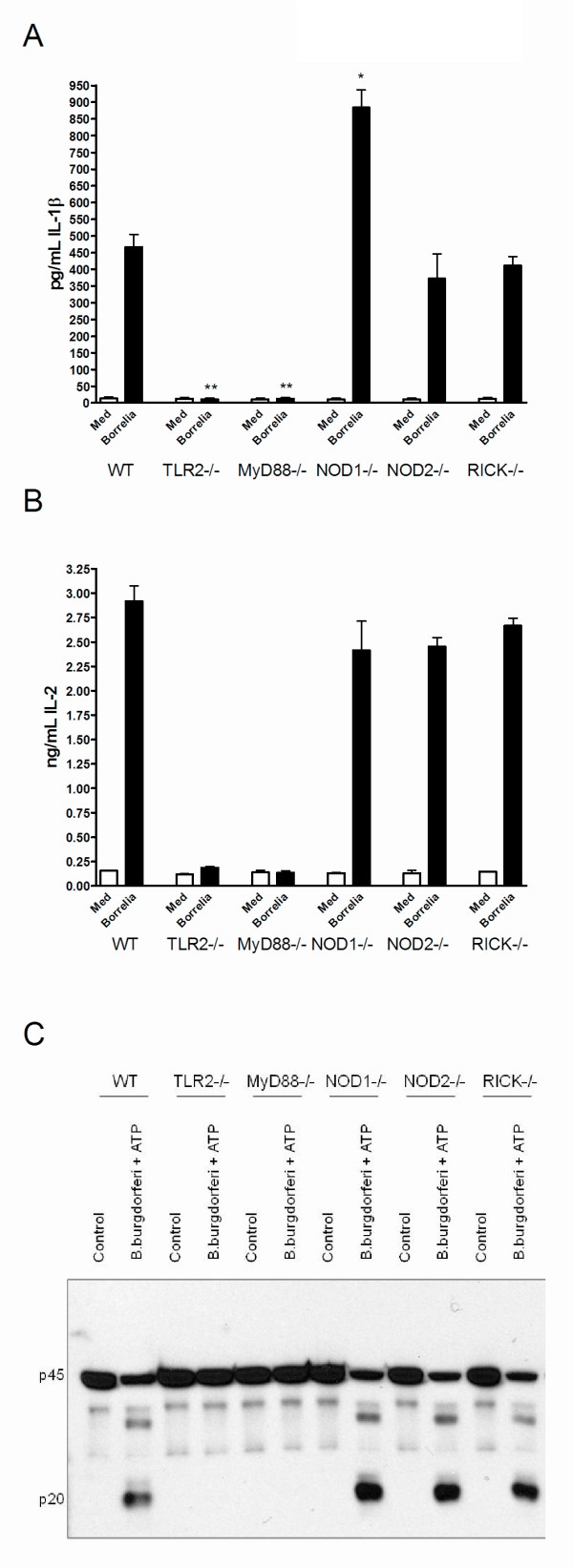
**TLR2 and MyD88 drive *Borrelia-*induced IL-1β production**. **(A) **0.5 × 10^6 Bone marrow-derived macrophages (BMDMs) from wild-type (WT), TLR2-/-, MyD88-/-, NOD1-/-, NOD2-/-, and RICK-/- mice were stimulated for 24 hours with either medium (Med), or 5 × 10^6 spirochetes per mL live *B. burgdorferi *(black bars). IL-1β protein levels in supernatant expressed in picograms per mL after BMDM exposure for 24 hours to *B. burgdorferi*. BMDMs are isolated from at least five animals per group, protein measurements were performed in duplicate. **(B) **IL-2 protein levels in nanograms per mL after BMDM stimulation with medium (Med), or 5 × 10^6 *B. burgdorferi *per mL for 24 hours, using cells from different knockout mice. **(C) **3 × 10^6 BMDMs from five WT C57Bl/6, TLR2-/-, MyD88-/-, NOD1-/-, NOD2-/-, or RICK-/- mice were incubated for 24 hours with or without 1 × 10^6 *B. burgdorferi *with adenosine triphosphate (ATP) (3 mM) for 30 minutes. Cleaved caspase-1 was detected by Western blotting using antibodies to detect the inactive caspase-1 (p45) or active caspase-1 (p20). The control conditions were already published in an earlier article by our group [19]. *Borrelia *and ATP by itself are unable to induce caspase-1 activation, the combination of the two are crucial for inducing cleavage. MyD88, myeloid differentiation factor 88; NOD, nucleotide-binding oligomerization domain; RICK, serine-threonine protein kinase with a caspase activation and recruitment domain; TLR, Toll-like receptor.

In humans, *B. burgdorferi-*induced IL-1β was partly NOD2-dependent, but the role of this PRR in the development of arthritis has never been demonstrated. The roles of murine NOD1, NOD2, and RICK were explored using mouse cells. BMDMs isolated from WT, NOD1-, NOD2-, or RICK-deficient mice were stimulated with either medium or *B. burgdorferi*. A clear induction of mRNA coding for IL-1β was seen in WT BMDMs upon stimulation with *B. burgdorferi *(Figure S1 in Additional file 2). Surprisingly, NOD1, NOD2, and RICK appeared not to be important for the induction of IL-1β after *B. burgdorferi *recognition, whereas - as expected - TLR2 and MyD88 were (Figure 2A). In the absence of NOD1, IL-1β mRNA as well as IL-1β protein levels were higher than in cells of WT mice, indicating an inhibitory role of NOD1 in *B. burgdorferi-*induced IL-1β production (Figure S1 in Additional file 2 and Figure S2A in Additional file 3). The IL-1 induced by BMDMs was bioactive in the IL-2 induction assay (Figure 2B). NOD1-, NOD2-, and RICK-deficient cells induced IL-6 and TNF-α production after exposure to *B. burgdorferi *(Figure S1 in Additional file 2).

### TLR2- and MyD88-mediated pathways are crucial for the *Borrelia*-induced IL-1β production

BMDMs isolated from WT, TLR2-/-, and MyD88-/- mice were exposed to either medium or *B. burgdorferi*. TLR2 and MyD88 are not only crucial for the induction of IL-1β-mRNA, but also important for the *B. burgdorferi-*induced IL-1β protein secretion in the supernatant (Figure S1 in Additional file 2 and Figure S2A in Additional file 3).

The IL-1β induced by BMDMs from WT mice was bioactive in the IL-2 assay, whereas no bioactive IL-1 production was seen by BMDMs of TLR2-/- and MyD88-/- mice (Figure 2B). In line with previous results, TLR2 and MyD88 are also critical for *B. burgdorferi-*induced IL-6 and TNF-α production on both protein and mRNA level (Figure S1B/C in Additional file 2).

### Caspase-1 activation by *B. burgdorferi *is dependent on TLR2 and MyD8

Inactive pro-caspase-1 needs to be cleaved to yield active caspase-1 before it can process pro-IL-1β. To identify the signaling cascades involved in *B. burgdorferi-*induced caspase-1 activation, BMDMs of TLR2-, MyD88-, NOD1-, NOD2-, or RICK-deficient mice were exposed to either medium (control) or *B. burgdorferi *plus adenosine triphosphate (ATP). Thereafter, Western blot analysis was performed using a specific antibody detecting the active subunit of caspase-1. WT cells stimulated with *B. burgdorferi *and ATP expressed the cleaved caspase-1 (Figure 2C). Surprisingly, activation of caspase-1 in BMDMs by *B. burgdorferi *is entirely dependent on TLR2- and Myd88-mediated pathways, whereas NOD1, NOD2, and RICK signaling pathways are not required for caspase-1 activation (Figure 2C).

### *B. burgdorferi *induces murine Lyme arthritis through TLR2 and MyD88

**Figure 3:**
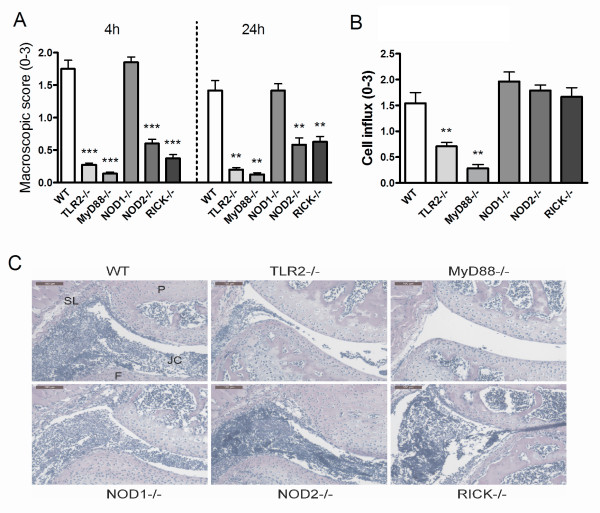
***B. burgdorferi-*induced murine Lyme arthritis is dependent on TLR2 and MyD88**. **(A) **Macroscopic score of the knees in wild-type (WT), TLR2 knockout (KO), MyD88 KO, NOD1 KO, NOD2 KO, and RICK KO mice. After 4 hours and 24 hours of intra-articular (i.a.) injection of 1 × 10^7 live *B. burgdorferi*, at least 10 knees per group. Data are mean ± SEM from 10 knees in each group; **P <0.01; ***P <0.0001, Mann-Whitney *U *test, two-tailed. **(B) **Scored cell influx 1 day after i.a. injection of *B. burgdorferi*. Data are mean ± SEM from 10 knees in each group; **P <0.01; Mann-Whitney *U *test, two-tailed. **(C) **Murine Lyme arthritis in WT, TLR2 KO, MyD88 KO, NOD1 KO, NOD2 KO, and RICK KO mice. Histology (H&E staining) 1 day after i.a. injection of *B. burgdorferi *in knee joints. 200× magnification; P, patella; F, femur; JC, joint cavity; SL, synovial lining. Scale bar represents 100 μM. MyD88, myeloid differentiation factor 88; NOD, nucleotide-binding oligomerization domain; RICK, serine-threonine protein kinase with a caspase activation and recruitment domain; TLR, Toll-like receptor.

As described before, both TLR2 and MyD88 play a critical role in *B. burgdorferi-*induced caspase-1 activation and subsequent IL-1β production *in vitro*. To assess the roles of these molecules *in vivo*, we induced Lyme arthritis by injecting live *B. burgdorferi *spirochetes into knee joints of WT, TLR2-, or MyD88-deficient mice. In addition, we investigated NOD1, NOD2, and RICK KO mice. Lyme arthritis, detected as joint swelling of the injected knee, could be clearly seen in WT mice. Both TLR2- and MyD88-deficient mice displayed significantly less joint swelling 4 h after induction of Lyme arthritis (Figure 3A). The lack of joint swelling was still noticeable after 24 h (Figure 3A). Interestingly, NOD1 KO mice displayed severe swelling, similar to that seen in WT mice. The expression of Lyme arthritis in NOD2 and RICK KO mice was significantly lower than in WT animals, both at early (4 h) and late (24 h) phases (Figure 3A). Next to joint swelling, the cell influx into the joint cavity at 24 h was assessed. In WT mice, the synovial lining was thickened and more cells, mainly neutrophils infiltrated into the joint cavity (Figure 3B and 3C). In both TLR2-, and MyD88-gene-deficient mice the synovial lining was less inflamed and significant reduced cell influx could be observed (Figure 3B and 3C).

### Inflammasome-mediated activation of caspase-1 by *B. burgdorferi*

**Figure 4:**
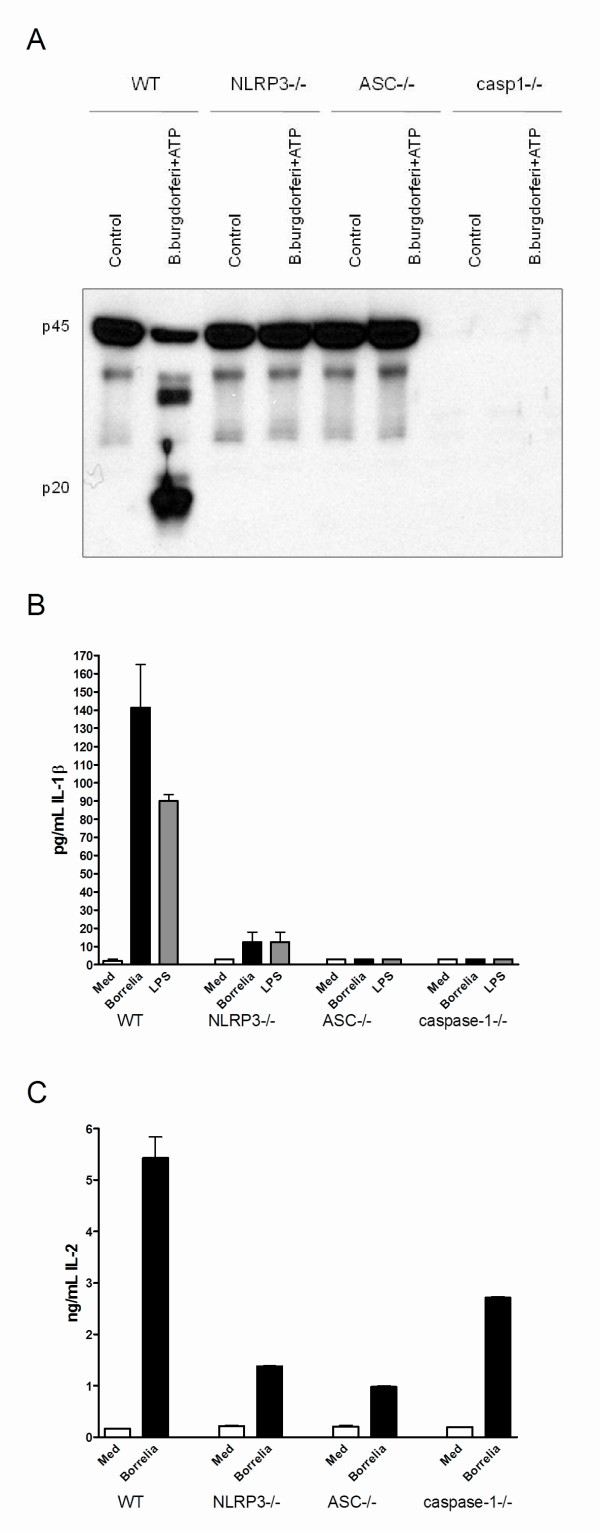
**Inflammasome components are necessary for *B. burgdorferi*-induced IL-1β**. **(A) **3 × 10^6 Bone marrow-derived macrophages (BMDMs) from wild-type (WT), NLRP3-, ASC-, and caspase-1 gene-deficient mice were stimulated for 24 hours with or without 1 × 10^6 *B. burgdorferi *with adenosine triphosphate (ATP) (3 mM) for 30 minutes. Cleaved caspase-1 was detected by Western blotting using antibodies to detect the inactive caspase-1 (p45) or active caspase-1 (p20). **(B) **IL-1β protein levels in the supernatant expressed in picograms per milliliter after BMDM exposure for 24 hours to *B. burgdorferi*, using WT, NLRP3-, ASC-, or caspase-1 gene-deficient mice. BMDMs are isolated from at least five animals per group, protein measurements were performed in duplicates. **(C) **IL-2 protein levels in nanograms per mL after BMDM stimulation with medium (Med), or 5 × 10^6 *B. burgdorferi *per mL for 24 hours, using cells from different knockout mice. ASC, apoptosis-associated speck-like protein containing a caspase recruitment domain (CARD); NLRP3, nucleotide oligomerization domain (NOD)-like receptor P3.

The roles of inflammasome components apoptosis-associated speck-like protein containing a caspase recruitment domain (CARD) (ASC), and NLRP3 in the *B. burgdorferi-*induced caspase-1 activation were assessed. Caspase-1 activation could be clearly detected when BMDMs of WT mice were exposed to *B. burgdorferi*, but was completely absent in cells from mice deficient in NLRP3, ASC, or caspase-1 (Figure 4A). Subsequently, NLRP3, ASC, and caspase-1 are crucial for total IL-1β protein production after *B. burgdorferi *stimulation (Figure 4B). Interestingly, a significantly decreased concentration of bioactive IL-1 could be observed in absence of ASC, NLRP3, or caspase-1, as compared to WT mice (Figure 4C). Transcription of IL-1β mRNA was not influenced by deficiency of the inflammasome components after *B. burgdorferi *stimulation of BMDMs (Figure S2A in Additional file 3). No differences in mRNA and protein levels of both IL-6 and TNF-α could be detected between WT, ASC-, NLRP3-, or caspase-1-deficient BMDM after stimulation with *B. burgdorferi *(Figure S2B/C in Additional file 3).

### Murine Lyme arthritis is ASC- and caspase-1-dependent, but NLRP3 independent

**Figure 5:**
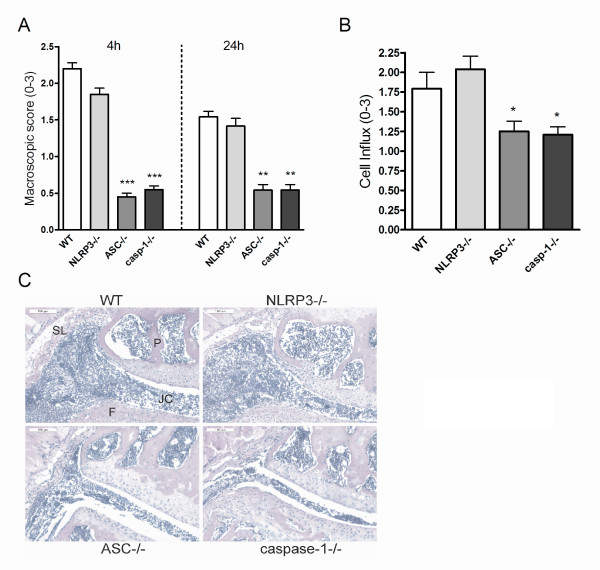
**ASC and caspase-1-dependent role in murine Lyme arthritis**. **(A) **Macroscopic score of the knees in wild-type (WT), NLRP3, ASC, and caspase-1 knockout mice. After 4 hours and 24 hours of intra-articular (i.a.) injection of 1 × 10^7 live *B. burgdorferi*, at least 10 knees per group. Data are mean ± SEM from 10 knees in each group; **P <0.01; ***P <0.0001; Mann-Whitney *U *test, two-tailed. **(B) **Scored cell influx 1 day after i.a. injection of *B. burgdorferi*. Data are mean ± SEM from 10 knees in each group; *P <0.05; Mann-Whitney *U *test, two-tailed. **(C) **Murine Lyme arthritis in WT, NLRP3, ASC, and caspase-1 knockout mice. Histology (H&E staining) 1 day after i.a. injection of *B. burgdorferi *in knee joints. 200× magnification; P, patella; F, femur; JC, joint cavity; SL, synovial lining. Scale bar represents 100 μM. ASC, apoptosis-associated speck-like protein containing a caspase recruitment domain (CARD); NLRP3, nucleotide oligomerization domain (NOD)-like receptor P3.

To assess the role of the inflammasome components *in vivo*, WT mice, NLRP3-/-, ASC-/-, and caspase-1-/- mice were injected with live *B. burgdorferi *and joint swelling was assessed after 4 h and 24 h (Figure 5A). ASC and caspase-1 knockout mice showed significantly reduced joint swelling after local *B. burgdorferi *injection, whereas NLRP3-gene-deficient mice displayed joint inflammation similar to WT animals (Figure 5A). ASC and caspase-1 KO mice had also less thickened synovial linings than WT animals, and less cell influx into the joint cavity could be observed in these mice (Figure 5B/C). Of interest, NLRP3 is not critical in the induction of murine Lyme arthritis.

### NLRP3-independent local cytokine production after *B. burgdorferi *injection

**Figure 6:**
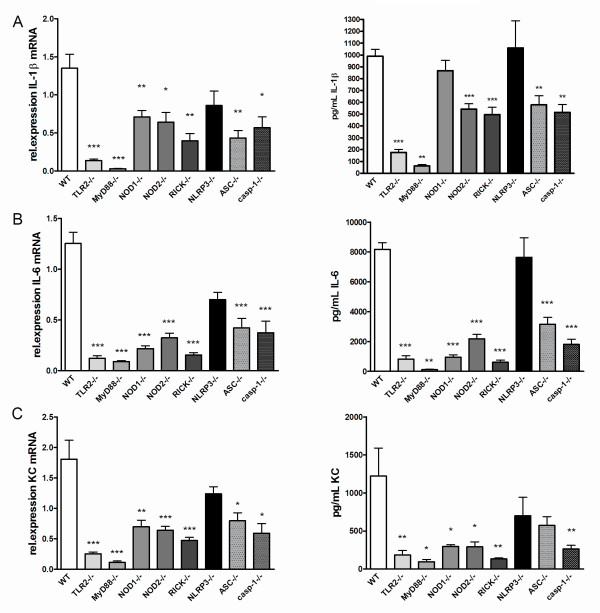
**TLR2- and MyD88-dependent and NLRP3 independent local cytokine production in synovium**. **(A) **IL-1β mRNA expression and protein levels, IL-6 mRNA expression and protein **(B)**, or keratinocyte-derived chemokine (KC) mRNA expression and protein **(C)**, in biopsies isolated from synovial tissues from either wild-type (WT), TLR2-, MyD88-, NOD1-, NOD2-, RICK-, NLRP3-, ASC-, and caspase-1 gene-deficient mice. Four hours after intra-articular (i.a.) injection with 1 × 10^7 live *B. burgdorferi*. At least five animals per group, bars represent mean ± SEM; *P <0.05; **P <0.01; ***P <0.0001, Mann-Whitney *U *test, two-sided. (A-C Right panels) Four hours after i.a. injection of 1 × 10^7 live *B. burgdorferi *in 10 mL of PBS, patellae from five WT C57BL/6 mice or knockout mice were cultured for 1 h and IL-1β (A), IL-6 (B), and KC (C) levels were measured using Luminex. Data are represented as mean ± SEM; five animals in each group; **P <0.01, ***P <0.001; Mann-Whitney *U *test, two-tailed. ASC, apoptosis-associated speck-like protein containing a caspase recruitment domain (CARD); MyD88, myeloid differentiation factor 88; NLRP3, NOD-like receptor P3; NOD, nucleotide-binding oligomerization domain; RICK, serine-threonine protein kinase with a caspase activation and recruitment domain; TLR, Toll-like receptor.

The strong reduction of arthritis was in line with the findings that the local cytokine production was ablated in both TLR2-, and MyD88-deficient mice (Figure 6). Synovial tissue explants revealed that both the mRNA expression as the protein production of IL-1β, IL-6, and KC was almost absent in TLR2 and MyD88 knockout mice (Figure 6A/C). A remarkable finding was the fact that NOD1, NOD2, and RICK signaling does not seem to be involved in the cell influx as similar numbers of inflammatory cells were found in the joint cavity as in WT mice (Figure 3B). This is in sharp contrast to the strongly reduced synovial production of IL-6, and KC in NOD1-/-, NOD2-/-, and RICK-/- mice, although NOD1-deficient mice produced similar amounts of IL-1β as WT mice and NOD2-/- and RICK-/- mice showed only 50% reduction in IL-1β (Figure 6). The inhibitory effect of NOD1 seen in Figure 3 for *Borrelia-*induced IL-1β production could not be observed using BMDM stimulation.

Next, we examined the local cytokine production in patellae after injection of *B. burgdorferi*. Levels of IL-1β, IL-6, and KC mRNA expression in synovial tissue of ASC and caspase-1-deficient mice was found to be significantly lower, with exception of NLRP3-deficient mice as compared to WT mice (Figure 6A). Similar results were observed for synovial IL-6 and KC mRNA expression (Figure 6B/C). Cytokine measurements in patella washouts showed that IL-1β production by the inflamed synovium was dependent on ASC and caspase-1, but not on NLRP3 (Figure 6A). This was also true for induction of IL-6, and KC (Figure 6B/C). Both ASC and caspase-1 are pivotal components for the induction of the latter cytokines.

## Discussion

In the present study, we describe for the first time the role of TLR2 and MyD88 *in vivo *in a murine model of Lyme arthritis elicited by local injection of *B. burgdorferi*. TLR2 and MyD88 are crucial for *B. burgdorferi-*dependent caspase-1 activation and subsequent IL-1β production *in vitro *by bone marrow-derived macrophages. Mice deficient in TLR2 or MyD88 display less joint swelling after i.a. injection of *B. burgdorferi *and fewer cells are attracted toward the joint cavity. In addition to TLR2 and MyD88, the inflammasome components ASC and caspase-1 are essential for IL-1β production by *B. burgdorferi-*stimulated BMDMs *in vitro*, and also are pivotal in the development of murine Lyme arthritis. The NOD1/NOD2/RICK pathway is also involved in synovial cell activation by *B. burgdorferi*, but is not essential for the production of IL-1β and for the influx of inflammatory cells into the joint cavity.

It is known that genetic background of mice influences the susceptibility for several experimental disease models [29,30]. This holds also true for the induction and maintenance of experimental murine Lyme disease. C3H/HeN mice are highly susceptible to develop severe arthritis upon intradermal injection with *Borrelia*. However, these C3H/HeN mice also display a defect in IL-12 production and therefore lack IL-12/IL-18-induced IFN-γ production upon stimulation with pathogens [31]. This is in contrast to cells from C57Bl/6 mice that produce IFN-γ after *Borrelia *exposure [19]. This might explain the susceptibility of the C3H/HeN mice to the development of Lyme arthritis. Lacking IFN-γ production results in less killing of invading *Borrelia *bacteria, resulting in dissemination through the body. In our study, we used C57Bl/6 mice, which do not display any defects in immune responses and therefore will reflect the disease development in humans more appropriately.

It has been suggested earlier [15], that IL-1 is a key player in the pathogenesis of Lyme arthritis. Patients with Lyme arthritis who were found to have elevated synovial concentrations of IL-1 Receptor antagonist (IL-1Ra) in combination with low concentrations of IL-1β showed a rapid resolution of Lyme arthritis. These data are in line with the results of the present study in murine Lyme arthritis. Mice deficient for the IL-1 receptor showed significantly attenuated *B. burgdorferi-*induced arthritis. In addition, decreased levels of IL-1β were noted in mice without functional IL-1R. Both mRNA expression and protein concentrations of IL-1β were lower in synovial cells lacking IL-1R, when compared to WT mice. These findings point towards an amplification loop in the local production of IL-1β via its receptor during the onset of Lyme arthritis.

Before active IL-1β can be secreted, the inflammasome needs to be assembled intracellularly by heteromultimerization [17]. NLRP3 is seen as one of the major NLRs that activates the inflammasome, followed by the cleavage of pro-caspase-1. In our hands, using caspase-1-deficient mice, caspase-1 seems to be the major enzyme to cleave pro-IL-1β. However, at later time points after *Borrelia *exposure, caspase-1 might play a different role. Liu *et al*., [32] showed that caspase-1 KO mice display higher arthritis scores at day 14 postinfection. On day 45, the arthritis scores in these mice are lower as compared to WT-type mice. However, the inoculation method between this study and our data differs and might cause a different amount of spirochetes in the joint at early time points. Next to this, recently it was demonstrated that the caspase-1 KO mice used in these studies were in fact double KO; they also lack functional caspase-11 [33]. Recently, it was demonstrated that mice deficient for only caspase-1 were more susceptible for intracellular infections [34]. The caspase-1/11 deficient mice were less susceptible, indicating that caspase-11 may dampen the effect of caspase-1 in our findings. Taken this together, caspase-1 plays an important role in the induction of an inflammatory response against *Borrelia *spirochetes in the joint, but is less important in controlling spirochete burden at later time points.

Here we show that *in vitro *activation of caspase-1 in BMDMs after *B. burgdorferi *exposure, as well as production of IL-1β protein and bioactive IL-1β, is indeed NLRP3-dependent (Figure 4). However, i.a. injection of *B. burgdorferi *did not differ between NLRP3 KO and WT mice in terms of joint inflammation and cytokine production. These results support recent reports showing that NLRP3 is not involved in other murine models of arthritis, such as antigen-induced arthritis, collagen-induced arthritis, or gouty arthritis [35-37]. We cannot fully explain the difference in NLRP3 dependency between the *in vitro *and *in vivo *induction of IL-1β by *B. burgdorferi*. Explanations may be sought in *in vivo *activation of additional inflammasomes, or triggering of ASC-caspase-1 independently of NLRs [38-40], but this remains to be demonstrated.

Interestingly, ASC was found to be pivotal in the induction of Lyme arthritis. It is known that ASC is not just an adaptor protein within the inflammasome, but also has a role in antigen presentation and lymphocyte migration. It addition, it has been demonstrated that ASC controls mRNA stability and expression of Dock2, which is involved in Rac activation in immune cells [41]. Finally, ASC has been associated with NFκB signaling [42], which is the major pathway in the production of several cytokines.

In contrast to TLR2-MyD88, neither NOD1 nor NOD2 were involved in the *B. burgdorferi*-triggered caspase-1 activation and bioactive IL-1β production in BMDMs (Figure 2B/C). However, *in vivo *we note a NOD1/NOD2/RICK-dependent cytokine production in synovial explants from mice with Lyme arthritis (Figure 6). The latter findings are in line with a recent report [3] in humans lacking functional NOD2, which show that *Borrelia-*induced IL-1β production is partly NOD2-dependent. Apparently, the recognition of *B. burgdorferi *by murine or human immune cells through PRRs differs. Although earlier results demonstrate a specific role for RICK and NOD2 in the recognition and induction of *Borrelia-*dependent IL-1β production by human cells, we were unable to detect the important role for NOD2 *in vivo *for cell influx in murine Lyme arthritis [3]. However, the role of these PRRs in the *in vitro *cytokine induction using murine cells could be confirmed in the present study.

An inhibitory role for NOD1 could be demonstrated in this manuscript, but was not found when studying human cytokine responses upon *Borrelia *stimulation. These discrepant observations might certainly be explained by differences between host species, it is described that humans express different PRR and explore different methods to induce a proper immune response against pathogens. Murine cells produce proinflammatory cytokines after *B. burgdorferi *exposure, such as IL-1β, KC, and TNF-α. Whether this difference is caused by hyperactive TLR2 signaling or lack of NOD2-mediated suppression is not elucidated yet.

The role of MyD88 in experimental Lyme disease, but also in the host response against *B. burgdorferi *was studied previously [43-45]. MyD88-deficient mice displayed severely higher amounts of spirochetes in several tissues, including the joints [11,43]. It was shown that MyD88 knockout mice infected with live *B. burgdorferi *displayed more severe arthritis and cell influx as compared to infected WT mice [43]. Therefore, it was concluded in this study that Lyme arthritis occurs without the presence of the MyD88 molecule. However, these findings can be explained by the fact that MyD88-deficient mice suffer from a higher spirochetal burden than WT mice. The excessive spirochetal load in the organs causes hyperinflammation that is MyD88-independent.

In the present study, we detected an important role for MyD88 in the development of early murine Lyme arthritis. Mice deficient in this adaptor molecule were not able to develop arthritis after i.a. injection of *B. burgdorferi*. TLR2 gene-deficient mice were also unable to induce Lyme arthritis at early time points after injection of spirochetes. These data suggest that TLR2-MyD88 signaling is very important during the onset of Lyme arthritis.

The treatment of patients suffering from Lyme arthritis is a challenge for clinicians, as treatment is often ineffective. Patients suffering from gout, rheumatoid arthritis, or other inflammatory joint diseases benefit from treatment with the IL-1 receptor antagonist (Anakinra) [46,47]. Based on present results indicating that IL-1 is one of the major mediators in Lyme arthritis, there is a rationale to propose that neutralizing IL-1 activity may also have beneficial effects in chronic Lyme arthritis. Apart from IL-1Ra, anti-IL-1β antibodies like Canakinumab might be useful for treatment of Lyme disease since these antibodies express a long half-life in humans. Thus, understanding the precise pathogenesis of Lyme disease may reveal novel therapeutic modalities in the near future.

## Conclusions

In the present study, we have demonstrated that murine Lyme arthritis is strongly dependent on IL-1 production. Next to that, murine Lyme arthritis is ASC and caspase-1-dependent, but NLRP3, NOD1, NOD2, and RICK independent. Caspase-1 activation by *B. burgdorferi *is dependent on TLR2 and MyD88.

In light of these findings, we propose a critical role for TLR2-MyD88-NLR-ASC-caspase-1 cascade-mediated IL-1β production in the pathogenesis of Lyme arthritis. The treatment of patients suffering from Lyme arthritis is still challenging for clinicians, and treatment for patients with chronic Lyme disease is often ineffective. Based on the present results indicating that IL-1 is one of the major mediators in Lyme arthritis, it is rationale to propose that neutralizing IL-1 activity may also have beneficial effects in chronic Lyme arthritis.

## Abbreviations

ASC: apoptosis-associated speck-like protein containing a CARD; ATP: adenosine triphosphate; BMDM: bone marrow-derived macrophages; BSK: Barbour-Stoenner Kelley; CARD: caspase recruitment domain; i.a., intra articular; IFN: interferon; IL: interleukin; KC: keratinocyte-derived chemokine; KO: knockout; MyD88: myeloid differentiation factor 88; NLR: NOD-like receptor; NOD: nucleotide-binding oligomerization domain; PCR: polymerase chain reaction; PRR: pattern recognition receptor; Ra: receptor antagonist; RICK: serine-threonine protein kinase with a caspase activation and recruitment domain; SNP: single nucleotide polymorphism; TLR: Toll-like receptor; TNF: tumor necrosis factor; WT: wild-type.

## Competing interests

The authors declare that they have no competing interests.

## Authors' contributions

MO, KB, RKSM, IV, MIK, FLV, TDK, and LABJ performed the experiments and drafted the manuscript. PS cultured and counted the *Borrelia *spirochetes. MIK performed the histology procedure. MO, KB, RKSM, PS, IV, MIK, FLV, JWMM, MGN, TDK, and LABJ critically revised the manuscript and approved the final manuscript.

## Supplementary Material

Additional file 1 **Supplemental materials and methods**. The missing information from the Materials and methods section. Click here for file

Additional file 2 **Figure S1. IL-1β mRNA expression is TLR2- and MyD88-dependent**. **(A) **IL-1β mRNA expression levels (x 1000) in bone marrow-derived macrophages isolated from wild-type (WT), TLR2-, MyD88-, NOD1-, NOD2-, and RICK gene-deficient mice. mRNA expression after 24 hours of stimulation with either medium or 5 × 10^6 *B. burgdorferi *per mL. At least five animals per group, bars represent mean ± SEM. *P <0.05; Mann-Whitney *U *test, two-tailed. IL-6 **(B) **and TNF-α **(C) **mRNA expression and protein production (in ng/mL for IL-6, and pg/mL for TNF-α, respectively) by bone marrow-derived macrophages isolated from WT, TLR2-, MyD88-, NOD1-, NOD2-, and RICK gene-deficient mice. mRNA expression after 24 hours of stimulation with either medium or 5 × 10^6 *B. burgdorferi *per mL. At least five animals per group, bars represent mean ± SEM. MyD88, myeloid differentiation factor 88; Nod, nucleotide-binding oligomerization domain; RICK, serine-threonine protein kinase with a caspase activation and recruitment domain; TLR, Toll-like receptor. Click here for file

Additional file 3 **Figure S2. Inflammasome-independent IL-1β transcription**. **(A) **IL-1β mRNA expression levels (× 1000) in bone marrow-derived macrophages isolated from wild-type (WT), NLRP3-, ASC-, and caspase-1 gene-deficient mice. mRNA expression after 24 hours of stimulation with either medium or 5 × 10^6 *B. burgdorferi *per mL. At least five animals per group, bars represent mean ± SEM. IL-6 **(B) **and TNF-α **(C) **mRNA expression and protein production (in ng/mL for IL-6, and pg/mL for TNF-α, respectively) by bone marrow-derived macrophages isolated from WT, NLRP3-, ASC-, and caspase-1 gene-deficient mice. mRNA expression after 24 hours of stimulation with either medium or 5 × 10^6 *B. burgdorferi *per mL. At least five animals per group, bars represent mean ± SEM. ASC, apoptosis-associated speck-like protein containing a caspase recruitment domain (CARD); NLRP3, nucleotide oligomerization domain (NOD)-like receptor P3. Click here for file
