# Supplementary material for: Murine Borrelia arthritis is highly dependent on ASC and caspase-1, but independent of NLRP3
Source: Arthritis Res Ther. 2012 Nov 13;14(6):R247. doi: 10.1186/ar4090 (PMC3674595; doi:10.1186/ar4090)
Supplement: Additional file 1 — Supplemental materials and methods. The missing information from the Materials and methods section. [file ar4090-S1.DOC]

**Additional file 1. Supplementary text, Materials and Methods section.**

***In vitro* cytokine production**

Bone marrow was isolated from dissected femurs and cultured in Iscove’s modified Dulbecco’s medium (IMDM) supplemented with 30% L929-conditioned medium containing macrophage-stimulating factor, 10% heat-inactivated fetal bovine serum (FBS), 5 mL non-essential amino acids (Invitrogen, Carlsbad, CA, USA), 50 µg/mL penicillin, and 50 µg/mL streptomycin at 37˚C in a humidified atmosphere containing 5% CO_2_. After three days of culture, new enriched IMDM medium was added. At day 5, bone marrow-derived macrophages (BMDMs) were collected using rubber scrapers and seeded at different concentrations and cultured an additional 24 hours before use. For stimulation of BMDMs, 0.5 x 10^6^ cells were seeded in a 96-well flat-bottom plate, and incubated for 24 hours at 37˚C in a humidified atmosphere containing 5% CO_2_. At the day of stimulation, culture supernatant was removed and cells were washed once with warm sterile phosphate-buffered saline (PBS, pH7.4). Thereafter, 0.2mL of either medium, live *Borrelia* spirochetes (5 x 10^6^/mL), or lipopolysaccharide (LPS) (10 µg/mL) plus 3 mM adenosine triphosphate (ATP) (last 30 minutes) was added to the cells as a stimulus, all made in medium without antibiotic additions. Cells were stimulated for 4 h and 24 h at 37˚C, 5% CO_2_.

**Cytokine measurements**

Mouse IL-1β, IL-6, keratinocyte-derived chemokine (KC), and TNFα concentrations were measured using Luminex (Bio-Rad, Hercules, CA, USA), according to the instructions of the manufacturer. Concentrations of mouse (intracellular) IL-1β were determined by specific radioimmunoassay (RIA; detection limit is 20 pg/mL) [49]. Levels of bioactive IL-1 was measured using a murine thymoma cell line EL4/NOB1 that produces IL-2 in response to active IL-1 as described before [50]. IL-2 levels were thereafter measured using commercially available ELISA (R&D Systems, Minneapolis, MN, USA; detection limit is 16 pg/mL).

**RNA isolation and real-time quantitative PCR**

Quantitative real-time PCR was performed using Power SYBR Green PCR Master Mix (Applied Biosystems, Carlsbad, CA, USA) using a 7300 Real-time PCR system (Applied Biosystems). In each PCR reaction, a melting curve analysis was included to control for a specific PCR amplification. Primers used for the experiments (final concentration 10 µM) are below. Real-time qPCR data were corrected for expression of the housekeeping gene mouse β-actin. Mouse IL-1β; Forward sequence 5’: GCA-ACT-GTT-CCT-GAA-CTC-AAC-T, Reversed sequence 5’: ATC-TTT-TGG-GGT-CCG-TCA-ACT. Mouse IL-6; Forward sequence 5’: CAA-GTC-GGA-GGC-TTA-ATT-ACA-CAT-G, Reversed sequence 5’: ATT-GCC-ATT-GCA-CAA-CTC-TTT-TCT. Mouse TNF-α; Forward sequence 5’: CAG-ACC-CTC-ACA-CTC-AGA-TCA-TCT, Reversed sequence 5’: CCT-CCA-CTT-GGT-GGT-TTG-CTA. Mouse β-actin; Forward sequence 5’: GGC-TGT-ATT-CCC-CTC-CAT-CG, Reversed sequence 5’: CCA-GTT-GGT-AAC-AAT-GCC-ATG-T. Cycling conditions were 2 min at 50°C and 10 min at 95°C followed by 40 cycli of 95°C for 15 sec and 1 min at 60°C.
