# Supplementary figures and images for: Murine Borrelia arthritis is highly dependent on ASC and caspase-1, but independent of NLRP3
Source: Arthritis Res Ther. 2012 Nov 13;14(6):R247. doi: 10.1186/ar4090 (PMC3674595; doi:10.1186/ar4090)

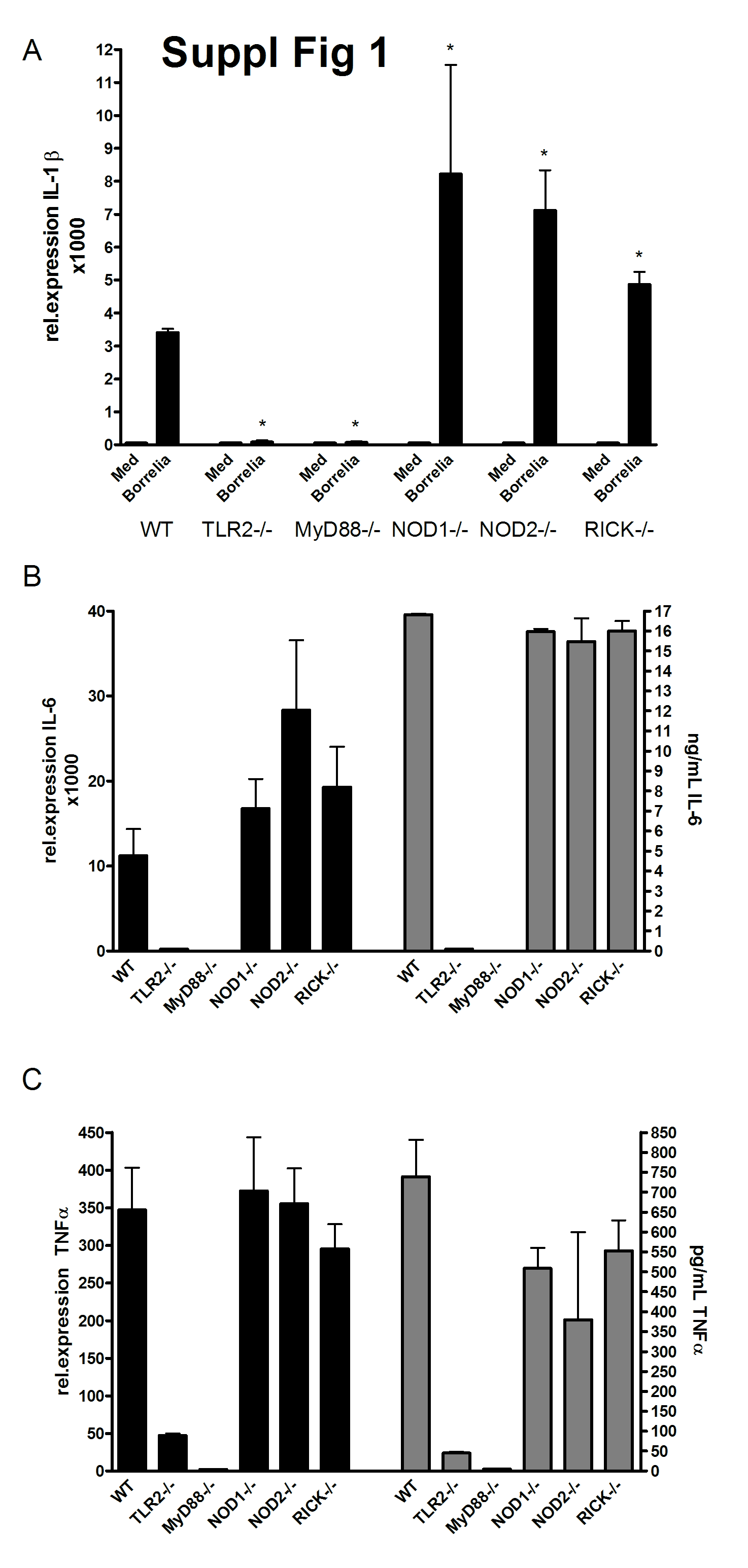

Supplement: Additional file 2 — Figure S1. IL-1β mRNA expression is TLR2- and MyD88-dependent. (A) IL-1β mRNA expression levels (x 1000) in bone marrow-derived macrophages isolated from wild-type (WT), TLR2-, MyD88-, NOD1-, NOD2-, and RICK gene-deficient mice. mRNA expression after 24 hours of stimulation with either medium or 5 × 10^6 B. burgdorferi per mL. At least five animals per group, bars represent mean ± SEM. *P <0.05; Mann-Whitney U test, two-tailed. IL-6 (B) and TNF-α (C) mRNA expression and protein production (in ng/mL for IL-6, and pg/mL for TNF-α, respectively) by bone marrow-derived macrophages isolated from WT, TLR2-, MyD88-, NOD1-, NOD2-, and RICK gene-deficient mice. mRNA expression after 24 hours of stimulation with either medium or 5 × 10^6 B. burgdorferi per mL. At least five animals per group, bars represent mean ± SEM. MyD88, myeloid differentiation factor 88; Nod, nucleotide-binding oligomerization domain; RICK, serine-threonine protein kinase with a caspase activation and recruitment domain; TLR, Toll-like receptor. [file ar4090-S2.TIFF]

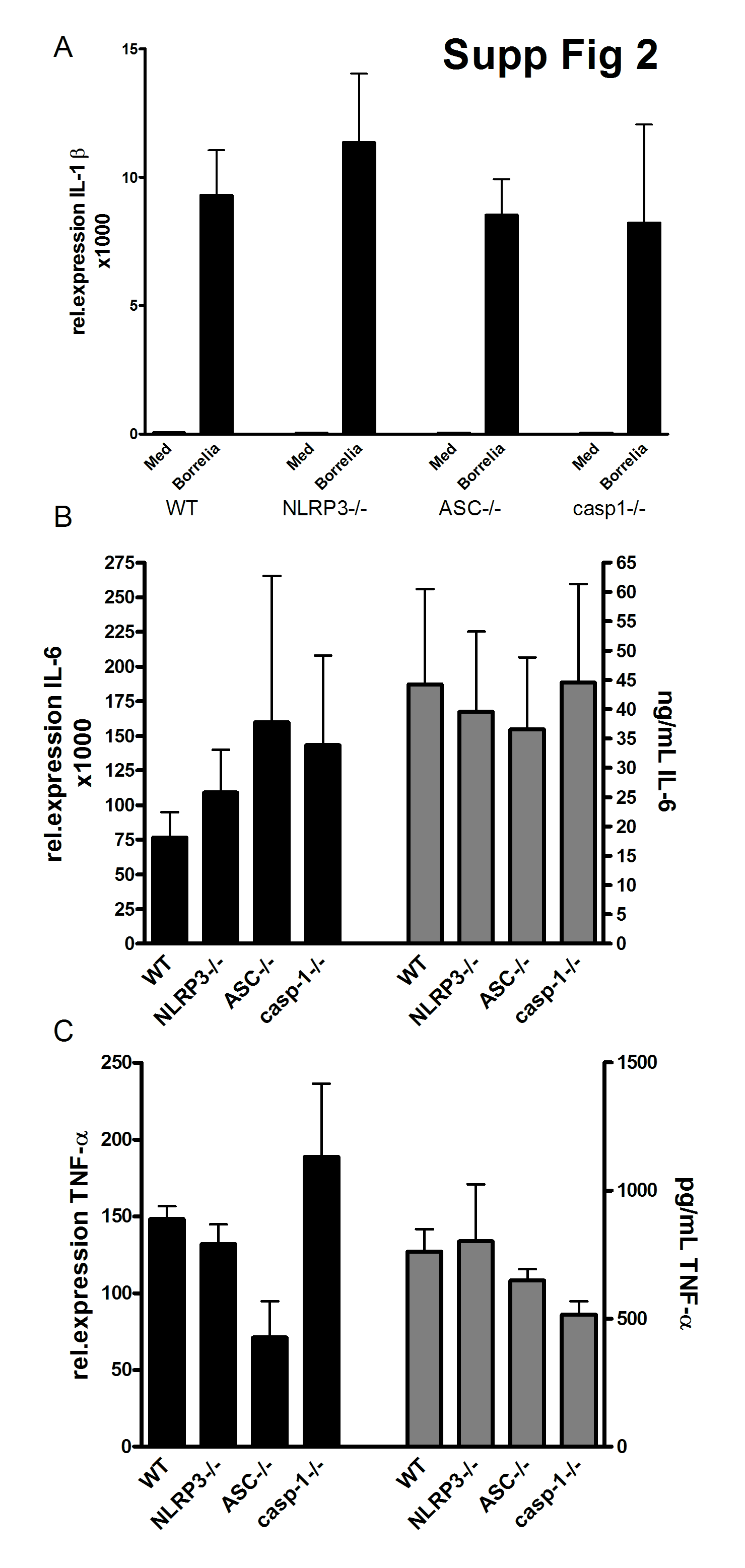

Supplement: Additional file 3 — Figure S2. Inflammasome-independent IL-1β transcription. (A) IL-1β mRNA expression levels (× 1000) in bone marrow-derived macrophages isolated from wild-type (WT), NLRP3-, ASC-, and caspase-1 gene-deficient mice. mRNA expression after 24 hours of stimulation with either medium or 5 × 10^6 B. burgdorferi per mL. At least five animals per group, bars represent mean ± SEM. IL-6 (B) and TNF-α (C) mRNA expression and protein production (in ng/mL for IL-6, and pg/mL for TNF-α, respectively) by bone marrow-derived macrophages isolated from WT, NLRP3-, ASC-, and caspase-1 gene-deficient mice. mRNA expression after 24 hours of stimulation with either medium or 5 × 10^6 B. burgdorferi per mL. At least five animals per group, bars represent mean ± SEM. ASC, apoptosis-associated speck-like protein containing a caspase recruitment domain (CARD); NLRP3, nucleotide oligomerization domain (NOD)-like receptor P3. [file ar4090-S3.TIFF]
